# Anticholinergic Burden of Geriatric Ward Inpatients

**DOI:** 10.3390/medicina57101115

**Published:** 2021-10-16

**Authors:** Krzysztof Wilczyński, Marta Gorczyca, Jagna Gołębiowska, Jan Szewieczek

**Affiliations:** 1 Department of Geriatrics, Faculty of Health Sciences in Katowice, Medical University of Silesia, Ziołowa 45/47, 40-635 Katowice, Poland; jszewieczek@sum.edu.pl; 2 Student Scientific Interest Group, Department of Geriatrics, Faculty of Medical Sciences, Medical University of Silesia, Ziołowa 45/47, 40-635 Katowice, Poland; marta486@vp.pl (M.G.); jagnagolebiowska@gmail.com (J.G.)

**Keywords:** ACB, anticholinergic medications, dementia, cognitive impairment, fall risk, functional disability

## Abstract

*Background and Objectives*: Anticholinergic drug use in the pharmacotherapy of elderly persons is common despite the increased risk of side effects. We examined the prevalence of anticholinergic drug use and total anticholinergic drug burden among patients admitted to an acute care geriatric ward in Poland. *Materials and Methods*: Cross-sectional study of 329 subjects hospitalized at the geriatric ward. Patient condition was assessed with a comprehensive geriatric assessment. The Anticholinergic Cognitive Burden (ACB) scale was used to estimate the total anticholinergic load. *Results*: Mean patient age was 79.61 ± 6.82 years. 40.73% of them were burdened with at least one anticholinergic drug. The clinically significant anticholinergic burden was observed in 13.98% of subjects. Patients with dementia, risk of falls, and severe disability had significantly higher total ACB scores compared to other groups. The receiver operating characteristics (ROC) curve revealed that the total ACB score ≥ 1 was significantly associated with dementia and the risk of falls. Total ACB score ≥ 2 was significantly associated with severe disability. *Conclusions*: Patients admitted to an acute care geriatric ward had an anticholinergic cognitive burden score comparable to other patient populations. We found associations at both low and elevated levels of anticholinergic burden with dementia and risk of falls. At elevated anticholinergic burden levels, we found associations with severe disability. Despite recommendations against the use of anticholinergics in older adults these medications are still commonly prescribed. Further study is necessary to define the characteristics of anticholinergic medication most closely associated with negative outcomes in elderly populations.

## 1. Introduction

Anticholinergic drugs (ACD) constitute a well-established pharmacological group with therapeutic benefits for neurological, genitourinary, respiratory [1], cardiac, gastrointestinal [2], and sleep [3] disorders. ACDs inhibit acetylcholine from acting on peripheral and central muscarinic receptors (M1, M2, M3, M4, and M5) [1]. Their clinical use is limited by significant side effects such as dry mouth, tachycardia, constipation, frequent urination, blurry vision, or reduced sudoriferous gland activity [1]. In addition, ACDs have been associated with cognitive decline, attention disorders, and confusion [1]. Such symptoms may especially aggravate the health of elderly persons and are especially troublesome for vulnerable senior-aged populations. This is most likely due to age-related physiological changes such as decreased acetylcholine production, increased permeability of the blood-brain barrier, and changes in pharmacokinetics and pharmacodynamics in the elderly [4,5,6]. As such, ACDs are more likely to have more significant side effects in old persons, and it is generally understood that ACDs should be avoided in these populations [7]. Our general clinical observations suggest that drugs with high anticholinergic properties, such as oxybutynin, solifenacin, scopolamine, and atropine, are more readily identified by physicians as affecting anticholinergic activity, while medicines characterized by low anticholinergic activity, like furosemide, metoprolol, digoxin, risperidone, haloperidol, and ranitidine are less readily identified as having anticholinergic properties [8]. However, ACDs, especially when combined together, may also exert a deleterious impact on the health status of vulnerable, frail old adults [8]. Previous studies have described associations between anticholinergic burden and the incidence of delirium in the elderly [9] as well as falls [10], cognitive impairment [2,11], and activities of daily living [12]. To systematically quantify the anticholinergic activity of medications used in clinical practice, several scales have been developed. For example, the Anticholinergic Cognitive Burden Scale (ACB), Anticholinergic Risk Scale (ARS), Anticholinergic Activity Scale (AAS), and Anticholinergic Drug Scale (ADS) [13].

Despite the widely recognized negative side effect profiles of anticholinergic drugs and their effect on the elderly, these medications are still commonly prescribed. While numerous studies have shown this phenomenon throughout the world, this problem has not been sufficiently examined in Poland. As such, our goal was to examine the anticholinergic burden of geriatric patients admitted to an acute care geriatric ward in Poland.

## 2. Materials and Methods

### 2.1. Design of Study, Participants and Measurements

A cross-sectional study of all consecutive patients (*n* = 329) admitted to the acute care geriatric ward at Leszek Giec Upper-Silesian Medical Centre of the Silesian Medical University in Katowice, Poland between March 2019, and October 2019, where a Comprehensive Geriatric Assessment (CGA) is completed for each admission. The CGA included patient history, physical examination, functional assessment, and ambulatory status. Data on the Barthel Index of Activities of Daily Living (Barthel Index) [14] was used to determine functional status. The Mini-Mental State Examination (MMSE) [15] was used to assess global cognitive performance. The Berg Balance Scale was used to assess the risk of falls. Increased fall risk was defined by Berg scores of ≤40 [16]. Barthel Index was used to evaluate the Activities of Daily Living and IADL for Instrumental Activities of Daily Living. Barthel Index scores range from 0 to 100, and MMSE scores range from 0 to 30; higher scores indicate better functional status. Barthel Index scores of 100–85 points were classified as good functional capacity, 84–20 points as moderately severe functional impairment, <20 points as severe disability [14]. Patient histories were analyzed for basic data such as ambulatory status. Dementia was diagnosed according to recommendations from the National Institute on Aging-Alzheimer’s Association [17]. Body mass index (BMI) was calculated for all patients.

### 2.2. Anticholinergic Cognitive Burden Scale

Patient anticholinergic burden before admission to the ward was calculated using the Anticholinergic Cognitive Burden (ACB) scale [18]. The ACB classifies drugs into 3 groups depending on their anticholinergic cognitive effect: 1—drugs with possible anticholinergic effect, 2 and 3—drugs with a clinical effect on cognition. The total ACB score is calculated by summation of each individual ACB drug score and reflects the cumulative patient anticholinergic burden.

### 2.3. Anticholinergic Medications

We divided patient medications according to the Anatomical Therapeutic Chemical Classification System into the following groups: antipsychotics (quetiapine, olanzapine, risperidone, haloperidol), anxiolytics (diazepam, hydroxyzine, alprazolam, clorazepate), antidepressants (trazodone, paroxetine, doxepin, venlafaxine, fluvoxamine) and cardiovascular medications (furosemide, chlorthalidone, captopril, digoxin, metoprolol, isosorbide, nifedipine).

### 2.4. Statistical Analysis

Data were analyzed using STATISTICA version 13 (StatSoft). Quantitative variables were presented as average values with standard deviation, categorical variables were shown as percentages. The χ2 test for categorical variables and the nonparametric Mann-Whitney U test for quantitative variables were used to compare individual groups with different levels of cognitive function, risk of falls, and activities of daily living. The ANOVA Kruskal-Wallis test was used to compare groups with different types of functional status. Spearman’s correlation coefficient was used to analyze correlations between ACB scores and results of MMSE, Barthel Index, and Berg Balance Scale. Normality was verified using the Shapiro-Wilk test. Using receiver operating characteristic (ROC) curves were aimed at finding an optimal cut-off of ACB scores predicted dementia incidence, severe disability, and risk of falls. The area under the ROC curve (AUC) defines the accuracy of this prediction. To analyze an association between values equal to cut-off points or greater and dementia incidence, severe disability, and risk of falls, univariate logistic regression was used, *p*-values < 0.05 were considered statistically significant.

### 2.5. Ethics

This study was conducted in accordance with the Declaration of Helsinki. The study protocol was registered with the Bioethical Committee of the Medical University of Silesia in Katowice, Poland. The project established that no procedure, including caregiver interview, exceeds standard procedures performed for patients hospitalized at the geriatrics department. The Bioethical Committee determined that in the context of law the study is not a medical experiment and does not require assessment by the bioethical committee (Letter KNW/0022/KB/236/19). Based on this decision, written informed consent of study participants was not required for our study, nor was separate patient consent required for our statistical analysis or research since patient data is not disclosed outside internal hospital ward staff. This decision enabled us to include patients with dementia for the study who would otherwise be excluded.

## 3. Results

### 3.1. Prevalence of Anticholinergic Burden

We examined 329 consecutive patients who were admitted to an acute care geriatric ward with an average age of 79.61 ± 6.82 years, 237 (72.04%) were women. Patients with an ACB score of 0 numbered 195 (59.27%), with an ACB score of at least 1 numbered 134 (40.73%), ACB score of 1 numbered 70 (21.28%), of 2 numbered 18 (5.47%), and with an ACB score greater than or equal to 3 numbered 46 (13.98%) (Table 1).

**Figure 1:**
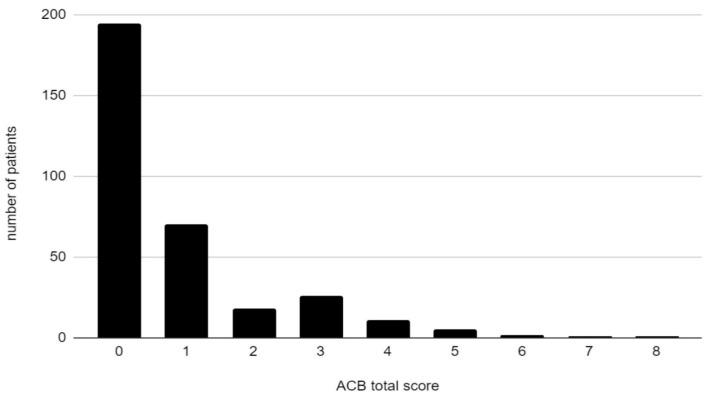
Total ACB score in the cohort.

Out of patients taking any drugs with anticholinergic activity, the most common score was 1 (70 patients), and the greatest ACB score was 8 (1 patient) (Figure 1).

The most common medications with anticholinergic activity were quetiapine (26 patients), metoprolol (24), furosemide (19), trazodone (17), diazepam (13), hydroxyzine (13), theophylline (11 persons), and digoxin (9).

### 3.2. Association of Anticholinergic Burden with Geriatric Disabilities

Our results show an inverse association between anticholinergic drug burden and cognitive function as measured by the MMSE and functional capacity as measured by the Barthel Index. ACB scores were associated with an increased risk of falls as measured by the Berg Balance Scale. ACB scores were negatively correlated with Barthel Index scores (*r* = −0.26, *p* < 0.0001), MMSE scores (*r* = −0.23, *p* = 0.0003), and Berg Balance Scale scores (*r* = −0.21, *p* = 0.0001). ACB scores were significantly higher in patients with dementia (1.26 ± 1.60) in comparison to older adults without dementia (0.50 ± 1.04) (*p* < 0.0001). Among persons with dementia, 23.38% had an ACB score of 3 or more (Table 2).

For elderly patients with a risk of falls (a Berg Balance Scale score of 40 points or less), the ACB index (1.06 ± 1.56) and the total number of drugs (9.28 ± 7.73) were significantly higher than in patients with a low risk of falls (a Berg Balance Scale score of 41 points or more) (0.57 ± 1.05, *p* = 0.004) (7.63 ± 4.45, *p* = 0.002) respectively. Among persons with a risk of falls, 18.18% (Berg Balance Scale score of 40 points or less) had an ACB score of 3 or more (Table 3).

Older adults with severe disability (Barthel Index score of <20 points) had significantly higher ACB scores (2.10 ± 2.30) than patients with moderately severe functional impairment (Barthel Index score of 84–20 points) (0.94 ± 1.33) (*p* = 0.037) and patients with good functional capacity (Barthel Index results of 85–100 points) (0.62 ± 1.20) (*p* = 0.012). Among subjects with severe disability (Barthel Index results of <20 points), 45% had an ACB score ≥ 3 (Table 4).

Among independently ambulating patients the ACB score was positively associated with a risk of falls (Berg Balance scale) (OR = 1.33, 95% CI 1.04–1.69, *p* = 0.021), but that relationship was not observed among patients requiring ambulation assistance.

**Figure 2:**
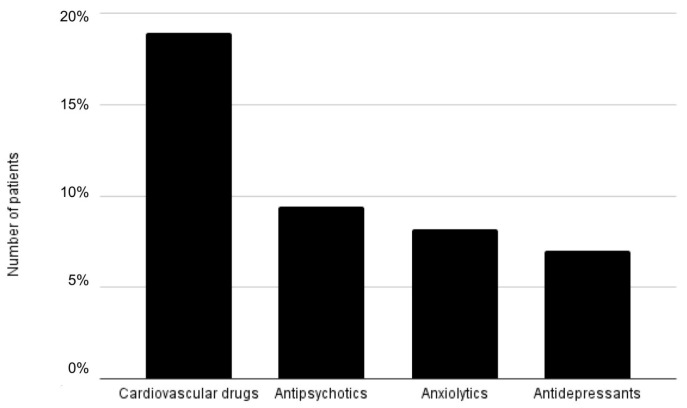
Distribution of anticholinergic medication groups among the cohort.

Cardiovascular drugs were the most common medication group taken by patients 18.90%, with approximately equal distribution of antipsychotics 9.40%, 8.20% anxiolytics, and antidepressants 7% (Figure 2).

### 3.3. Cut-Off Point of ACB Score Significantly Associated with Geriatric Problems

**Figure 3:**
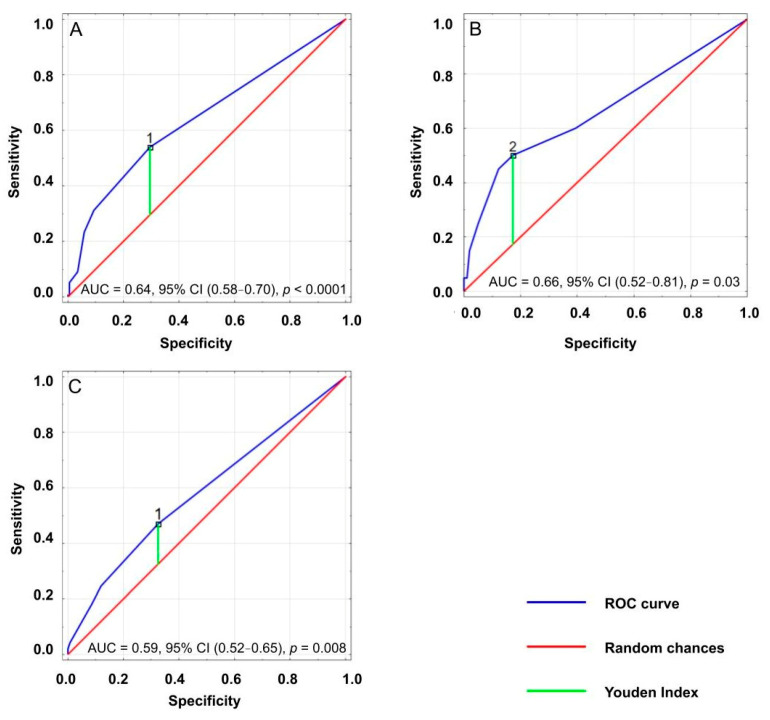
Receiver operating characteristics (ROC) curve analysis for ACB scores in relation to dementia incidence (**A**), severe disability (**B**) and risk of falls (**C**) in geriatric patients.

Analysis of the receiver operating characteristic (ROC) curves showed significant cut-off points of total ACB scores for dementia incidence, risk of falls and severe disability. These cut-off points were equal to 1 for dementia incidence and risk of falls, and 2 for severe disability (Figure 3) (Table 5). Univariate logistic regression performed based on cut-off points revealed a positive association between an ACB score of at least 1 and dementia incidence and risk of falls. Severe disability was associated with an ACB score of at least 2 (Table 6).

## 4. Discussion

We examined the anticholinergic burden of medication used by patients admitted to an acute care geriatric ward. To our knowledge, this is the first such study in Poland to examine this important group of patients. We also did not find any other study that examined the association of ACB drug burden with dementia, fall risk, and functional capacity in patients admitted to an acute care geriatric ward.

The prevalence of anticholinergic drug use in our cohort seems to be comparable to other population studies of anticholinergic medication prevalence among the elderly. In 2019 Green et al. examined a cohort of elderly persons with mild cognitive impairment or dementia (*n* = 10,698) who were part of a comprehensive managed care system in the USA (Kaiser Permanente Colorado) for the use of anticholinergic drugs. They reported that 63% of the cohort were treated with at least 1 drug with ACB [10]. A study conducted in Slovenia by Cebron Lipovec et al. in 2020 [19] on elderly outpatients reported that 43.2% were treated with at least 1 ACB drug. Additionally, in 2017 Pfistermeister et al. in Germany [20] examined patients admitted to geriatric wards and reported that 46.3% were being treated with at least 1 ACB drug. In a study of 341 community-dwelling elderly in the USA, West et al. reported that persons who had total ACB scores greater or equal to 3 made up 47.8% of the cohort [21], while Cebron Lipovec et al. in 2020 reported that this same subset constituted 12.1% of their cohort [20]. Vaughan et al. reported that 37.9% of hospitalized patients with dementia had ACB ≥ 3 or were prescribed one medication with an ACB of 2 or higher [22].

As expected, and in line with similar studies, we found associations between anticholinergic drug use and decreased cognitive function [23], functional capacity [24], and increased fall risk [25]. Importantly, the degree of anticholinergic burden of medications taken by elderly persons seems to be a factor in the degree of patient disability. Studies have shown that the strongest correlation of negative clinical outcomes is with drugs with significant anticholinergic activity. Less clear in the literature is the association of anticholinergics with weak anticholinergic activity. Campbell et al. in 2010 showed an association between increasing anticholinergic burden and increased risk of cognitive impairment [11]. A study by Pfistermeister et al. also reported a positive association between total anticholinergic cognitive burden and cognitive impairment in patients hospitalized in geriatric wards [20]. In 2019 Coupland et al. reported that in a cohort of primary care patients exposed to several types of drugs with significant anticholinergic activity such as antidepressants or antipsychotics is associated with an increased risk of dementia [26]. A similar study by Richardson in 2018 reported a positive association between antidepressant medications with ACB scores of 1 and 3 and dementia incidence, but there was no association between antipsychotics with ACB scores of 3 and dementia in the elderly [2]. Both studies included the elderly with dementia and a control group. In 2020 Naharci et al. found that among community-dwelling elderly, the use of at least one medication with possible or defined anticholinergic properties was associated with falls in frail and pre-frail subjects [27]. Green et al. showed that a combination of drugs with ACB scores of 2 and 3 are associated with a higher risk of falls in the elderly [10]. Additionally, greater ACB scores were shown to be a risk factor for falls or fractures in adults with overactive bladder [28]. In 2012 Pasina et al. [12] showed that elderly who were treated with anticholinergic medications had lower Barthel Index scores than patients not treated with anticholinergics. Boccardi et al. in 2017 reported that impairment in Activities of Daily Living was more prevalent among users of anticholinergics, independently of their cognitive status [24]. Lockery [29] et al. showed that older persons with ACB scores greater or equal to 3 were associated with dementia compared to persons with anACB load of 0 and that the risk of dementia was similar when comparing patients with ACB scores of 1 and 2. Boustani et al. suggest that the total anticholinergic burden of an elderly patient with mild cognitive impairment, cognitive impairment, dementia, or delirium should be maintained below 3, and medications with ACB activity of 2 and 3 should be removed or replaced with medications with lower ACB scores where possible [8]. Our results suggest clinically relevant adverse associations with low anticholinergic medications. Based on the work of others and our own study, it seems that further study is necessary for more granular quantification of anticholinergic medication factors that may affect the clinical outcomes of elderly persons.

That anticholinergic medication should be avoided in elderly patients is commonly accepted in the medical community and described in guidelines such as the Beer’s criteria [7]. Despite this, our results show that 13.68% of elderly patients who are admitted to an acute care geriatric ward are still prescribed drugs with high anticholinergic activity which can significantly affect their functional and mental status [10,26]. Reasons for increased anticholinergic burden among the elderly are likely multifactorial but include the following factors: inherent multimorbidity of advanced age [30], failure to recognize anticholinergic medication side effects that lead to prescription cascade [31], and also the reluctance of prescribers to remove medications prescribed by other prescribers [32]. While general guidelines are well defined, evidence-based clinical recommendations for substitution of drugs with strong anticholinergic activity are not well-defined in the literature. From our clinical experience, sometimes good alternatives to drugs with anticholinergic activity are not available. While there exists considerable evidence of associations between anticholinergic burden and adverse outcomes, proof of a direct benefit of reducing medication-related anticholinergic burden is lacking in the literature. Our own empirical clinical experience in the acute care geriatric ward suggests that reducing anticholinergic burden improves clinical outcomes in elderly patients. However, Yeh et al. examined the potential benefits of reducing medication-related anticholinergic burden for a small population of older adults with dementia and the results were inconclusive [33]. In addition, Kersten et al. in 2013 described interventions that significantly reduced ACB but did not appreciably improve cognitive function in nursing home residents [34]. However, a more recent study in 2019 showed that ACB reduction among elderly subjects admitted to an acute care unit was associated with a reduction in neuropsychiatric symptoms and caregiver burden [35]. To our knowledge, no studies have explicitly examined the benefit of ACB reduction by replacement of medications with lower ACB counterparts. It seems that further research is needed for definitive evidence of improvement in the context of anticholinergic burden drug substitution. Further study also seems necessary to define the characteristics of anticholinergic medication most closely associated with negative outcomes in elderly populations.

Limitations of our study include lack of detailed information on medication compliance, and we did not analyze medication dosage. This may be of significance as anticholinergic symptoms may manifest at higher levels of drug concentrations. However, as preliminary results, our study demonstrates risk for anticholinergic medication regardless of dosage. Several factors may account for the differences in anticholinergic prevalence among our elderly population and other studies: different levels of dementia advancement, education, age, and ethnicity. In addition, patients referred to an acute geriatric ward will be, by definition, burdened with greater morbidity and possibly, as a result, burdened, with increased drug use. However, the impact of anticholinergic drug use cannot be discounted in hospitalized patients and future work may focus on the isolation of potentially confounding factors to definitely assess the impact of anticholinergic drug use on this particularly vulnerable population

## 5. Conclusions

Patients admitted to an acute care geriatric ward had an anticholinergic cognitive burden score comparable to that of other patient populations described in the literature. We found associations at both low and elevated levels of anticholinergic burden with dementia and risk of falls. At elevated anticholinergic burden levels, we found associations with severe disability. Despite recommendations against the use of anticholinergics in older adults these medications are still commonly prescribed.

## Figures and Tables

**Table 1:** Cohort characteristics. Data shown as mean values and standard deviation ($\bar{x}$ ± SD) for quantitative variables and percentages for categorical variables.

Variable	Total Cohort *n* = 329
ACB (score)	0.85 ± 1.38
ACB score of at least 1 (%)	40.73
ACB score of 0 (%)	59.27
ACB score of 1 (%)	21.28
ACB score of 2 (%)	5.47
ACB score of at least 3 (%)	13.98
Medications with an ACB score ≥2 (%)	13.68
Sex, women (%)	72.04
Age (years)	79.61± 6.82
Total number of drugs	8.59 ± 6.54
BMI (kg/m^2^)	27.96 ± 5.67
Systolic blood pressure (mmHg)	140.87 ± 21.66
Diastolic blood pressure (mmHg)	81.44 ± 12.42
Independently ambulating (%)	57.85
Ambulatory with assistance (%)	34.77
Nonambulatory (%)	7.38
Barthel Index (score)	73.64 ± 26.93
IADL Index (score)	18.92 ± 6.52
Berg Balance Scale (score)	33.26 ± 16.66
MMSE (score)	22.05 ± 6.97
Dementia (%)	47.68
Previous stroke (%)	8.23
Diabetes (%)	30.89

Abbreviations: ACB score—anticholinergic cognitive burden score; BMI-body mass index; IADL Index-Instrumental Activities of Daily Living Scale; MMSE score-Mini Mental State Examination.

**Table 2:** Characteristics of anticholinergic burden in patients with and without dementia. Data shown as mean values and standard deviations ($\bar{x}$ ± SD ) for quantitative variables and percentages for categorical variables.

Variable	Patients with Dementia *n* = 154	Patients without Dementia *n* = 169	*p* Value
ACB (score)	1.26 ± 1.60	0.50 ± 1.04	<0.001
ACB score = 0 (%)	46.10	70.41	<0.001
ACB score = 1 (%)	22.73	20.12	0.397
ACB score = 2 (%)	7.79	3.55	0.149
ACB score ≥ 3 (%)	23.38	5.92	<0.001
Any medication with an ACB score ≥2 (%)	22.73	5.92	<0.001

Abbreviations: *n*, number of subjects; ns, non-significant; ACB score, anticholinergic cognitive burden score.

**Table 3:** Characteristics of anticholinergic burden in patients with different risk of falls assessed by Berg score. Data shown as mean values and standard deviations ($\bar{x}$ ± SD) for quantitative variables and percentages for categorical variables.

Variable	Patients with a Risk of Falls *n* = 187	Patient with Low Risk of Falls *n* = 126	*p* Value
ACB (score)	1.06 ± 1.56	0.57 ± 1.05	0.004
ACB score = 0 (%)	52.94	67.46	0.010
ACB score = 1 (%)	22.46	20.63	0.744
ACB score = 2 (%)	6.42	3.17	0.407
ACB score ≥ 3 (%)	18.18	8.73	0.011
Any medications with ACB score ≥2 (%)	17.65	8.73	0.026

Abbreviations: *n*, number of subjects; ns, non-significant; ACB score, anticholinergic cognitive burden score.

**Table 4:** Characteristics of anticholinergic burden in patients with different functional status. Data shown as mean values and standard deviations ($\bar{x}$ ± SD) for quantitative variables and percentages for categorical variables.

Variable	Patients with Severe Disability *n* = 20	Patients with Moderately Severe Functional Impairment *n* = 142	Patients with Good Functional Capacity *n* = 160	*p* Value
ACB (score)	2.10 ± 2.30	0.94 ± 1.33	0.62 ± 1.20	<0.001
ACB score = 0 (%)	40.00	51.41	68.75	0.002
ACB score = 1 (%)	10.00	26.06	18.75	0.123
ACB score = 2 (%)	5.00	8.45	1.88	0.054
ACB score ≥ 3 (%)	45.00	14.08	10.63	<0.001
Any medication with an ACB score ≥2 (%)	40.00	14.08	10.63	0.001

Abbreviations: *n*, number of subjects; ns, non-significant; ACB score, anticholinergic cognitive burden score.

**Table 5:** Optimal cut-off points of total ACB scores for dementia incidence, severe disability and risk of falls.

Outcomes	Cut-Off Value	Sensitivity	Specificity	Youden Index	AUC	*p* Value
Dementia	1	0.54	0.30	0.24	0.64	<0.001
Severe disability	2	0.50	0.17	0.33	0.66	0.026
Risk of falls	1	0.47	0.33	0.15	0.59	0.008

**Table 6:** Univariate logistic regressions performed to reveal associations between outcomes and ACB scores values equal to or greater than the optimal cut-off point by ROC curve. Data shown as odds ratio (OR), 95% confidence interval (95% CI).

Outcomes	OR	95% CI	*p* Value
Dementia	2.86	1.80–4.52	<0.001
Severe disability	4.81	1.90–12.18	<0.001
Risk of falls	1.88	1.17–3.02	0.008

## Data Availability

Not applicable.
